# Harnessing semiochemicals for parasitoid-based biological control: from laboratory identification to field applications

**DOI:** 10.1007/s44297-025-00061-4

**Published:** 2025-11-30

**Authors:** Hao Guo, Chen-Zhu Wang

**Affiliations:** 1 College of Life Science, Institute of Life Science and Green Development, Hebei University, Baoding, China; 2 Shanxi Key Laboratory of Nucleic Acid Biopesticides, School of Synthetic Biology, School of Life Science, Shanxi University, Taiyuan, China; 3 State Key Laboratory of Animal Biodiversity Conservation and Integrated Pest Management, Institute of Zoology, Chinese Academy of Sciences, Beijing, China

**Keywords:** Parasitoids, Semiochemicals, Tritrophic interactions, Biological control, Synthetic biology

## Abstract

Parasitoids are vital biological control agents in agricultural pest management, with mating and parasitism as their core behaviors essential for reproduction and survival. In recent decades, advanced analytical techniques, such as gas chromatography-electroantennographic detection (GC-EAD), have enabled the identification of key semiochemicals that regulate parasitoid behavior. Notably, studies on *Campoletis chlorideae* (Hymenoptera: Ichneumonidae) have elucidated the mechanisms of sex pheromone communication, advancing our understanding of pheromonal signaling in parasitoids. Moreover, plant-derived synomones and host-derived kairomones serve as pivotal chemical cues for host location, underpinning tritrophic (plant–pest–parasitoid) interactions. The functional characterization of olfactory receptors tuned to herbivore-induced plant volatiles and kairomones, achieved through ectopic expression systems, has further clarified the molecular mechanism underlying semiochemical-mediated behaviors. Synthetic biology offers promising avenues for manipulating parasitoid behavior by leveraging genetic and metabolic engineering of plants and yeast to release critical synomones and kairomones, thereby improving parasitoid recruitment. This review synthesizes the role of semiochemicals in mediating parasitoid behaviors, evaluates methodologies for behavioral manipulation, and explores the potential and limitations of integrating synthetic biology with semiochemicals to advance sustainable pest management.

## Introduction

Parasitoids, a specific insect family comprising more than 100,000 described species, are predominantly found within the orders Hymenoptera (approximately 78%), Diptera (approximately 20%), and, to a lesser extent, Coleoptera, Lepidoptera, and Neuroptera [1–4]. Unlike true parasites, which permit host survival, parasitoids cause host mortality during development. These lethal interactions position parasitoids as critical components of biological control strategies targeting agricultural and environmental pests. For example, species of the genus *Trichogramma* (Hymenoptera: Trichogrammatidae) parasitize the eggs of hundreds of insect species, particularly Lepidoptera [5]. In the Yellow River and Yangtze River Basins in China, the ichneumonid wasp *Campoletis chlorideae* (Hymenoptera: Ichneumonidae) parasitizes and kills up to 40% of *Helicoverpa armigera* larvae [6].

The earliest documented record of insect parasitism dates to 1096 AD in China, where Lu Dian described the life cycle of a tachinid fly and its oviposition on silkworm caterpillars [7]. In the seventeenth century, Italian naturalist Antonio Vallisneri provided one of the first scientific accounts of parasitoid‒host interactions, detailing the relationship between the parasitoid *Apanteles glomeratus* (Hymenoptera: Braconidae) and its host, the cabbage butterfly *Pieris rapae* (Lepidoptera: Pieridae) [8]. The deliberate use of biotic agents for controlling insect and weed pests emerged as a modern practice approximately 1890. A landmark event was the successful intercontinental introduction of *A. glomeratus* from England to California in 1883 to suppress invasive *P. rapae*, followed by subsequent introductions of parasitoids, such as *Cryptochetum iceryae* (Diptera: Cryptochaetidae) from Australia in 1888, *Scutellista cyanea* (Hymenoptera: Pteromalidae) from South Africa in 1901, and *Ephialtes caudatus* from Spain in 1904 to the United States, for controlling pests such as *Laspeyresia pomonella* [9]. In 1907, researchers at the University of Kansas collected and distributed large numbers of *Aphidius testaceipes*-parasitized aphids (*Schizaphis graminum*) to the aphid-occurring area to increase parasitism, marking an early effort to leverage native parasitoids for biological pest control [9]. By 1900, advancements in techniques and facilities for managing natural enemies facilitated the design and implementation of biological control programs across multiple regions and continents. The commercial application of parasitoids began in the 1920s, with the chalcid wasp *Encarsia formosa* (Hymenoptera: Aphelinidae) being employed to control the greenhouse whitefly *Trialeurodes vaporariorum* (Homoptera: Aleyrodidae) in European greenhouses, pioneering the inundative release of parasitoids for augmentative biological control [10]. These early successes established parasitoids as vital tools in integrated pest management (IPM), with ongoing advancements shaping their use in modern agriculture.

Contemporary biology control is implemented through three primary approaches: (1) classical biological control, involving the import and establishment of exotic species; (2) augmentative biological control, which involves mass rearing and periodic release of established species (indigenous or exotic); and (3) conservation biological control, which focuses on preserving natural enemies through environmental manipulation [11]. Among these, augmentative biological control has garnered significant attention from biocontrol practitioners. A prominent example is the mass rearing and release of the egg parasitoid *Trichogramma* Spp., with efficient, commercially developed rearing systems worldwide [5, 12–16]. Other successful cases of augmentative biological control by mass rearing and release include *Cotesia* spp. [17] and *Telenomus* spp. [18, 19]. The successful release of parasitoids for biocontrol depends on the synchronization of parasitoid deployment with the vulnerable life stages of the pests. Two main release approaches are employed: inoculative release, which aims to re-establish or bolster natural enemy populations that have declined due to unfavorable conditions, and inundative release, which seeks immediate pest suppression through large-scale releases [20].

Following release or emergence, parasitoids rely on semiochemicals, also known as infochemicals, derived from the host habitat and hosts to locate suitable mates and hosts, a process critical for successful mating and parasitism [21–26]. These processes are guided by a sequence of steps influenced by environmental cues that vary in detectability and reliability [22, 27, 28]. Plant volatiles, which are emitted from host habitats, are abundant and highly detectable but less reliable, whereas sex pheromones, herbivore-induced plant volatiles, and host-derived compounds are more reliable indicators of mate and host presence, accessibility, and suitability, although they are hard to detect. In recent decades, numerous semiochemicals associated with the mating and host location of parasitoids have been identified. This review synthesizes the role of semiochemicals in mediating mating and parasitism behaviors in parasitoids, with a focus on the behavioral regulatory potential and practical applications of key compounds.

## Sex pheromone and mate localization

The species-specific nature of sex pheromones is critical for mate recognition in insects. Since the identification of bombykol, the first insect sex pheromone, in the silkworm moth [29], research on insect sex pheromones and their perception mechanisms has been a cornerstone of chemical ecology. Sex pheromones in Lepidoptera are well studied and typically include unsaturated aldehydes, alcohols, and esters with 10–18 carbon atoms [30]. In contrast, research on parasitoid sex pheromones is less advanced, primarily because of their small body size and low pheromone release rates, resulting in limited molecular and physiological evidence available [31]. In parasitoids, sex pheromones are predominantly released by females and are perceived by males [32, 33]. For example, in 1970, Assem described the courtship behavior of male *Lariophagus distinguedus* (Hymenoptera: Pteromalidae), which includes wing vibration, orientation, mounting, and antennal and head movement in response to female cues [34].

The volatile sex pheromones of parasitoids include aldehydes, ketones, esters, and olefins, which have diverse structures, some of which feature complex methyl side chains or benzene rings (Table 1). Recently, tetradecanal and 2-heptadecanone (in a 1:4.6 ratio) were identified as sex pheromones released by female *C. chlorideae*, a parasitoid of the cotton bollworm. When these compounds were applied at this ratio to n-hexane-washed female wasps, they restored male attraction and elicited characteristic courtship wing-fanning behavior [48].

**Table 1:** Reported Airborne sex pheromones of parasitoids

Parasitoids	Sex pheromones	Resources	Methods	References
*Itoplectis* *conquisitor*	Geranial; Neral	Anhydrous ether extracts of female bodies	GC‒MS; Cage preference	[35]
*Ascogaster* *reticulatus*	(*Z*)−9-hexadecenal	Hexane extracts of female bodies	GC‒MS; Mate localization	[36]
*Macrocentrus* *grandii*	(*Z*)−4-tridecenal	Hexane extracts of female bodies	GC‒MS; Wind tunnel; Field test	[37]
*Ascogaster* *quadridentata*	(*Z*,*Z*)−9,12-octadecadienal	Porapak Q collection of female body volatiles	GC-EAD; GC‒MS; Y-tube olfactometer; Field test	[38]
*Melittobia* *digitata*	(*E*)-bergamotene	Diethyl ether extracts of male bodies; SPME collection of male volatiles	GC‒MS; Preference test	[39]
*Nasonia* *vitripennis*	(4*R*,5*R*)−5-hydroxy-4-decanolide; (4*R*,5*S*)−5-hydroxy-4-decanolide; 4-methylquinazoline	Dichloromethane extracts of male abdomens	GC‒MS; Four-chamber olfactometer	[40]
*Cephalonomia* *tarsalis*	Dodecanal	Dichloromethane extracts of female bodies	GC‒MS; Four-chamber olfactometer	[41]
*Spalangia endius*	Methyl 6-methylsalicylate	SPME and SuperQ collection of female volatiles	GC‒MS; GC-EAD; Mate localization	[42]
*Leptopilina* *heterotoma*	(-)-iridomyrmecin	Dichloromethane extracts of female bodies	GC‒MS; Y-tube olfactometer; Mating assay	[43, 44]
*Leptopilina ryukyuensis*	(-)-iridomyrmecin	Dichloromethane extracts of female bodies	GC‒MS; Mating assay	[44]
*Leptopilina* *japonica*	(-)-iridomyrmeci	Dichloromethane extracts of female bodies	GC‒MS; Mating assay	[44]
*Cotesia glomerata*	Nonanal	Dichloromethane extracts of female and male bodies	GC-EAD; Four-arm olfactometer	[45]
*Cotesia marginiventris*	Heptanal	SuperQ collection of female volatiles; Dichloromethane extracts of female bodies	GC-EAD; GC-PFC; Four-arm olfactometer	[46]
*Tetrastichus planipennisi*	(6*S*,10*S*)-(2*E*,4*E*,8*E*)−4,6,8,10- tetramethyltrideca-2,4,8-triene	HayeSep-Q collection of female volatiles	GC‒MS; NMR; Wind tunnel; Field test	[47]
*Campoletis chlorideae*	Tetradecanal; 2-heptadecanone	Hexane extraction of female bodies	GC-EAD; GC‒MS; Y-tube olfactometer; Mating assay	[48]
*Microplitis mediator*	*n*-octyl acrylate	Hexane extraction of female bodies	GC-EAD; GC‒MS; Y-tube olfactometer; Cage experiment; Mating assay	[49]

*GC‒MS* gas chromatography‒mass spectrometry, *GC‒EAD* gas chromatography‒electroantennographic detection, *SPME* solid phase microextraction

In addition to female-produced pheromones, some parasitoids use male-released pheromones to attract females. For example, male *Nasonia vitripennis* (Hymenoptera: Pteromalidae) release (4*R*,5*R*)−5-hydroxy-4-decanolide and (4*R*,5*S*)−5-hydroxy-4-decanolide to attract females, marking the first identified male sex pheromones in parasitoids [40] (Table 1). In addition to volatile sex pheromones, cuticular hydrocarbons (CHCs) serve as contact pheromones in parasitoids. In *Dibrachys cavus* (Hymenoptera: Pteromalidae), 3-methylnonacosane and 3-methylhentriacontane on the cuticle of virgin females act as contact pheromone components [50]. Similarly, 7-methyltetracosane and 3-methylheptacosane have been identified as contact pheromone components in *Urolepis rufipes* (Hymenoptera: Pteromalidae) and *Lariophagus distinguendus* (Hymenoptera: Pteromalidae), respectively [51, 52]. Owing to differences in volatility, volatile pheromones and CHCs likely function at different stages of courtship: volatile pheromones mediate long-distance mate searching, whereas CHCs provide critical information about mate suitability at close range. To date, no single parasitoid species has been systematically studied for both volatile and contact pheromones, likely owing to methodological limitations. Future studies should focus on concurrent investigations of volatile and contact sex pheromones to elucidate the full spectrum of sex pheromone communication mechanisms in parasitoids.

## Host searching and localization

Host localization by parasitoids involves two primary stages: (1) long-distance host habitat searching and (2) close-range host localization. During these processes, foraging parasitoids navigate complex volatile environments and face a reliability‒detectability dilemma [53, 54]. When searching for host habitats, parasitoids rely on abundant but less reliable environmental cues, such as plant volatiles. In contrast, pinpointing host presence requires more specific semiochemicals, such as herbivore-induced synomones and host-derived kairomones, which are highly reliable but less detectable from a distance.

### Host habitat searching

Upon emergence, parasitoids face the challenge of locating host habitats in unfamiliar environments, a task that becomes particularly critical when host population densities are high or host availability is low, prompting dispersal to new habitats [55]. In nature, host habitats emit diverse plant-derived volatile compounds, which are highly detectable by parasitoid olfactory systems and guide them over long distances [56]. However, plant volatiles are less reliable indicators, as they only suggest the potential presence of hosts. Visual cues, such as variations in leaf shape, may provide supplementary information, but olfaction remains the primary sensory modality [57]. Both female and male parasitoids exploit host plant odors to locate habitats, although their objectives differ: females search for hosts for oviposition, whereas males seek females for mating within these habitats. Additionally, both sexes may share a common goal of locating food sources, such as nectar and pollen [58]. Recent research highlights the role of microbial emissions in influencing parasitoid behavior. For example, odors from nectar fermented by bacteria (*Staphylococcus epidermidis*, *Terrabacillus saccharophilus*, *Pantoea* sp., and *Curtobacterium* sp.) isolated from buckwheat *Fagopyrum esculentum* are attractive to the egg parasitoid *Trissolcus basalis* (*Hymenoptera: Scelionida*e) [59]. Moreover, styrene and benzaldehyde, which are emitted by bacterial strains isolated from the habitat of the generalist aphid parasitoid *Aphidius colemani* (Hymenoptera: Aphidiinae), are attractive to this parasitoid [60]. Taken together, parasitoids integrate multiple habitat-associated olfactory cues, including plant volatiles and microbiota missions, to orient themselves toward host habitats.

### Host localization

Upon arriving at the host habitat, the primary task of female parasitoids is to locate suitable hosts [61]. Within the diverse biotic communities of host habitats, females typically search within 1–2 cm of host-associated plants, using antennae and other chemosensory organs to probe plant tissues damaged by hosts [61, 62]. Chemical cues for host localization are diverse but can be categorized into two types: (1) “distress signals” released by plants in response to host damage and (2) kairomones emitted by the host, such as sex pheromones, aggregation pheromones, fecal odors, or scales. These compounds are released in low quantities but exhibit high species specificity, making them less detectable but highly reliable. As parasitism is a female-specific behavior, females are thought to display greater sensitivity to herbivore-induced plant volatiles (HIPVs) and host kairomones, whereas males exhibit reduced sensitivity [63].

#### Plant-derived synomones

##### Herbivore-induced plant volatiles (HIPVs)

HIPVs serve as synomones for parasitoids, guiding parasitoids to find concealed hosts [64, 65]. Since the 1980s, researchers have recognized that plants damaged by herbivores emit volatile compounds to recruit parasitoids for indirect defense [66]. Studies from the 1980s to the 1990s on tritrophic interactions (plant–herbivore–parasitoid) established that volatiles from pest-damaged plants are species specific, providing reliable cues for host localization. This led to the formal recognition of HIPVs as key semiochemicals mediating tri-trophic interactions in the early 1990s [67, 68]. The composition of HIPVs varies among plant species and even within the same plant species, depending on the herbivore involved [24, 65]. HIPVs primarily consist of six-carbon green leaf volatiles (GLVs), aliphatic compounds, terpenoids, and aromatic compounds [24]. Mass spectrometry analyses comparing volatiles from healthy and pest-damaged plants have revealed HIPV profiles for crops, such as cotton [69–73], maize [68, 74–77], tobacco [78–80], kidney bean [81], and tomato [82]. Common HIPV components include green leaf volatiles such as (*Z*)−3-hexen-1-ol and (*Z*)−3-hexenyl acetate; monoterpenes such as (*E*)-*β*-ocimene and linalool; sesquiterpenes such as (*E*,*E*)-*α*-farnesene and (*E*)-*β*-caryophyllene; and homoterpenes such as (*E*)−4,8-dimethyl-1,3,7-nonatriene (DMNT) and (*E*,*E*)−4,8,12-trimethyl-1,3,7,11-tridecatetraene (TMTT) [24, 83]. Although HIPVs comprise multiple components, parasitoids may respond to only a subset, underscoring the importance of identifying active HIPV components to increase parasitoid efficacy in pest control. To date, research has focused primarily on the chemical identification of HIPVs and parasitoid behavioral responses to HIPV blends, with few studies exploring the regulatory effects of individual components on parasitoid behavior. Table 2 summarizes the reported HIPV components that significantly attract parasitoids or increase their parasitism efficiency.

**Table 2:** Active components of herbivore-induced plant volatiles

Tritrophic interactions	Effective volatiles	Methods	Effects	References
Cotton-*Helicoverpa armigera*-*Campoletis chlorideae*	(*Z*)−3-hexenyl acetate; (*Z*)-jasmone	Olfactometer; Cage experiment; Field test	Attracting females; Enhancing parasitism	[69, 84, 85]
*Phaseolus vulgaris*- *Trialeurodes vaporariorum*- *Encarsia formosa*	(*Z*)−3-hexen-1-ol; 3-octanone; DMNT	GC‒MS; Wind tunnel	Attracting females	[81]
Maize- *Mythinma separata*-* Campoletis chlorideae*	(*Z*)−3-hexenyl acetate; Linalool	GC‒MS; Olfactometer	Attracting females	[77]
*Fabaceae* spp.-*Liriomyza huidobrensis*-*Opius dissitus*	(*Z*)−3-hexen-ol	GC‒MS; Olfactometer	Attracting females	[86]
Cotton-*Helicoverpa armigera*- *Microplitis mediator*	Nonanal; DMNT; (*Z*)−3-hexenyl acetate	GC-EAD; Olfactometer; Cage experiment	Attracting females; Enhancing parasitism	[87]
Maize-*Chilo partellus*- *Trichogramma bournieri*	(*E*)-(1R,9S)-caryophyllene; DMNT; TMTT	GC‒MS; Olfactometer	Attracting females	[88]
Litchi- *Tessaratoma papillosa-Anastatus japonicus*	β-caryophyllene; Undecane; (*E*)-α-farnesene (+)-aromadendrene; (*Z*)−3-hexen-ol	GC‒MS; Oviposition Preference assay	Attracting females; Facilitating host egg localization	[89]

DMNT: (3*E*)−4,8-dimethyl-1,3,7-nonatriene; TMTT: (*E*,*E*)−4,8,12-trimethyl-1,3,7,11-tridecatetraene (TMTT); GC‒MS: gas chromatography‒mass spectrometry; GC‒EAD: gas chromatography‒electroantennographic detection

Recent advances in sequencing technologies have enabled the identification of chemosensory-related genes in parasitoids, paving the way for "reverse chemical ecology" to identify active components of HIPVs. For example, in the parasitoid *C. chlorideae*, transcriptome sequencing revealed that an odorant receptor, *CchlOR62*, is exclusively expressed in female antennae [84]. Functional studies demonstrated that CchlOR62 responds to (*Z*)-jasmone, an HIPV component found in cotton and tobacco. Behavioral assays confirmed that (*Z*)-jasmone strongly attracts female parasitoids and significantly enhances their parasitism efficiency in early-instar *H. armigera* larvae. These findings highlight reverse chemical ecology as a rapid and reliable approach for identifying bioactive components of HIPVs [84].

##### Oviposition-induced plant volatiles (OIPVs)

Recent studies have demonstrated that oviposition by herbivorous insects, either alone or in combination with feeding, induces the emission of oviposition-induced plant volatiles (OIPVs) that function as synomones, particularly those attracting egg parasitoids [90–92]. For example, oviposition by the pine sawfly *Diprion pini* (Hymenoptera: Diprionidae) on *Pinus sylvestris* needles triggers volatile emissions that attract the egg parasitoid *Chrysonotomyia ruforum* (Hymenoptera: Eulophidae) [93]. Similarly, volatiles emitted by bean plants following oviposition by the bug *Nezara viridula* (Heteroptera: Pentatomidae) strongly attract the egg parasitoid *Trissolcus basalis* (Hymenoptera: Scelionidae) [94]. In maize landraces, oviposition by the stemborer *Chilo partellus* (Lepidoptera: Pyralidae) induces the emission of volatiles, including DMNT, TMTT, and (*E*)-(1*R*,9*S*)-caryophyllene, which significantly attract the egg parasitoid *Trichogramma bournieri* and the larval parasitoid *Cotesia sesamiae* (Hymenoptera: Trichogrammatidae) in four-arm olfactometer tests [88]. In tomato plants, oviposition by *Tuta absoluta* (Lepidoptera: Gelechiidae) induces volatile emissions that attract the parasitoid *Trichogramma achaeae* (Hymenoptera: Trichogrammatidae) [95]. Similarly, oviposition by* H. armigera* on tomato plants triggers the release of α-pinene, which is attractive to *Trichogramma chilonis* (Hymenoptera: Trichogrammatidae) [96]*.* Additionally, oviposition by *Spodoptera litura* (Lepidoptera: Noctuidae) on tobacco plants induces the emission of linalool, tetracosane, and (*Z*)−3-hexenyl acetate, which attract the egg parasitoid *Telenomus remus* (Hymenoptera: Scelionidae) [97]. Recent research has also indicated that OIPVs convey precise information about egg age. For example, the egg parasitoid *Trichogramma japonicum* (Hymenoptera: Trichogrammatidae) strongly influences the odor of rice plants bearing 2-day-old eggs of the rice leaf folder *Cnaphalocrocis medinalis* (Lepidoptera: Pyralidae) over plants with younger or older eggs, which is mediated by the detection of the OIPV components D-limonene and *α*-pinene [98]. These findings highlight the role of OIPVs as key synomones that mediate host location by egg parasitoids.

##### Pathogen-induced plant volatiles (PIPVs)

Plant pathogen infections induce the release of volatile bouquets that attract insect vectors, facilitating pathogen transmission [99–101]. Emerging evidence suggests that parasitoids exploit these pathogen‒plant‒insect vector interactions by eavesdropping on pathogen‒induced plant volatiles to locate their hosts. For example, *Cotesia glomerata* (Hymenoptera: Braconidae) exhibits a stronger preference for volatiles emitted by aphid-infested plants infected with the compatible pathogen *Xanthomonas campestris* pv. *campestris* compared with those infected with an incompatible pathogen or healthy plants [102]. Similarly, *Cotesia marginiventris* (Hymenoptera: Braconidae) is more attracted to fungus-infected peanut plants attacked by *Spodoptera exigua* (Lepidoptera: Noctuidae) than to non-infected ones [103]. Infection of pepper plants (*Capsicum annuum* cv. Yolo Wonder) with cucumber mosaic virus (CMV) or potato virus Y (PVY) increases their attractiveness to the parasitoid *A. colemani* [104]. Moreover, bacterial infection of orange *Citrus sinensis* seedlings by *Candidatus* Liberibacter asiaticus induces the emission of methyl salicylate, which attracts the parasitoid *Tamarixia radiata* (Hymenoptera: Eulophidae) and significantly enhances parasitism of the pest *Diaphorina citri* (Homoptera: Psyllidae) [105]. Conversely, some plant pathogens do not influence or may negatively affect parasitoid behavior. For example, maize seedlings infected by the fungus *Setosphaeria turcica* do not alter the behavior of the parasitoids *C*. *marginiventris* or *Microplitis rufiventris* (Hymenoptera: Braconidae) [106]. Similarly, red-rot infection in sugarcane reduces the attractiveness of sugarcane borer-induced volatiles to the parasitoid *Cotesia flavipes* (Hymenoptera: Braconidae) [107]. These findings indicate the complexity of pathogen-mediated effects on plant volatile emissions and parasitoid behavior, which warrants further investigation to elucidate the underlying mechanisms and ecological implications.

#### Host-derived kairomones

##### Host pheromones

Insect sex pheromones are critical for mate attraction due to their high species specificity, providing a reliable cue for their natural enemies, including parasitoids. In particular, owing to the proximity of the ovipositor to the sex pheromone gland, residual sex pheromones may be deposited on eggs or plant tissues during oviposition, further assisting parasitoids in host location [108]. For example, both *T. chilonis* and *Cotesia plutellae* (Hymenoptera: Braconidae), the egg parasitoids of the diamondback moth *Plutella xylostella* (Lepidoptera: Plutellidae), showed significant attraction to a blend of the moth’s sex pheromones—(*Z*)−11-hexadecenal, (*Z*)−11-hexadecenyl acetate, and (*Z*)−9-hexadecenol (1:1:0.01)—in Y-tube olfactometer assays [109]. Consistently, in Y-tube olfactometer assays, *Telenomus busseolae* (Hymenoptera: Scelionidae) was significantly attracted to the sex pheromone blend of its host, *Sesamia nonagrioides* (Lepidoptera: Noctuidae), during the calling period, consisting of (*Z*)−11-hexadecenyl acetate, (*Z*)−11-hexadecenol, (*Z*)−11-hexadecenal, and dodecyl acetate (8.5:1:1:2) [110]. Field trapping experiments demonstrated that *Telenomus euproctidis* (Hymenoptera: Scelionidae) was strongly attracted to the primary sex pheromone component of its host, *Euproctis taiwana* (Lepidoptera: Lymantriidae), (*Z*)−16-methyl-9-heptadecenyl isobutyrate [111]. Similarly, *Telenomus podisi* (Hymenoptera: Scelionidae) was significantly attracted to the sex pheromone methyl 2,6,10-trimethyltridecanoate released by male *Euschistus heros* (Heteroptera: Pentatomidae) in a polypropylene cage (24 cm × 24 cm × 8 cm) [112]. Moreover, four-arm olfactometer assays revealed that *Chrysonotomyia ruforum* (Hymenoptera, Eulophidae) was attracted to the primary sex pheromone components of its hosts, *D*. *pini* and *Neodiprion sertifer* (Hymenoptera: Diprionidae), specifically (2*S*,3*R*,7*R*)−3,7-dimethyl-2-tridecyl acetate and (2*S*,3*S*,7*S*)−3,7-dimethyl-2-pentadecyl acetate, respectively, with this preference being unaffected by prior parasitic experience [113].

Intriguingly, larval parasitoids also respond to host sex pheromones. Reportedly, insect sex pheromones can persist in the environment for days, with degradation rates varying significantly on the basis of abiotic factors such as temperature, UV radiation, and humidity [114]. Additionally, sex pheromones can be adsorbed onto plant tissues, such as leaves, which may further slow the degradation of sex pheromones, providing a temporal window for parasitoids to detect these residual cues and locate host larvae [115, 116]. In wind tunnel experiments, the larval parasitoid *Leptopilina heterotoma* (Hymenoptera: Eucoilidae) exhibited a strong preference for the sex pheromone of its host, *Drosophila melanogaster* (Diptera: Drosophilidae), specifically (*Z*)−11-vaccenyl acetate (cVA), which consistently selects cVA-containing lures in cage experiments [117]. Similarly, *Wroughtonia ligator* (Hymenoptera: Braconidae) preferentially selected sticky traps baited with the sex pheromone component of its host, *Neoclytus acuminatus acuminatus* (Coleoptera: Cerambycidae), (2,3)-hexanediol, in field experiments [118]. The ectoparasitoid *Bracon hebetor* (Hymenoptera: Braconidae), which targets pyralid moths, exhibited electrophysiological and behavioral responses to the male sex pheromones of *Galleria mellonella* (Lepidoptera: Pyralidae), nonanal and undecanal (7:3), in gas chromatography-electroantennographic detection (GC-EAD) and Y-tube olfactometer experiments, with responses observed in mated but inexperienced females [119]. Field and laboratory choice experiments demonstrated that the sex pheromones of *Planococcus ficus* (Hemiptera: Pseudococcidae), (*S*)-( +)-lavandulyl senecioate, attracted its parasitoid *Anagyrus pseudococci* (Hymenoptera: Encyrtidae) [120]. The sex pheromone component of *Myzus persicae* (Homoptera: Aphididae), (4*aS*,7*S*,7*aR*)-nepetalactone elicited strong electrophysiological responses in the antennae of its parasitoid *A. colemani* and induced prolonged residence behavior [121]. Likewise, electroantennogram analysis further revealed that *C. chlorideae* exhibited strong electrophysiological responses to the sex pheromone components of its host,* H. armigera*, (*Z*)−11-hexadecenal and (*Z*)−9-hexadecenal; however, whether sex pheromones attract parasitoids awaits further investigation [122]. Moreover, *a microplitis mediator* (Hymenoptera: Braconidae) exhibited chemotaxis toward the sex pheromone component of its host, *H. armigera*, (*Z*)−9-tetradecenal, in a Y-tube olfactometer [123].

In addition to sex pheromones, other host pheromones, including alarm, aggregation, and trail pheromones, serve as host-finding kairomones for parasitoids. For example, the aphid alarm pheromone (*E*)-*β*-farnesene acts as a host-finding kairomone for *Aphidius uzbekistanicus* (Hymenoptera: Aphidiidae), *Aphidius ervi* (Hymenoptera: Aphidiidae), and *Diaeretiella rapae* (Hymenoptera: Braconidae) [124–126]. The specialist parasitoid *Pseudacteon tricuspis* (Diptera: Phoridae), which targets the red imported fire ant, uses alarm pheromones released by worker ants to orient and locate its host [127]. This parasitoid also exhibits significant electrophysiological and behavioral responses to 2,6-dialkylpiperidines, which are alkaloid components of the venom of red fire ants, indicating their role as olfactory cues for host localization [128]. *Apocephalus paraponerae* (Diptera: Phoridae), a specialist parasitoid of the ant *Paraponera clavate* (Hymenoptera: Formicidae), utilizes two mandibular gland alarm pheromones, 4-methyl-3-heptanone and 4-methyl-3-heptanol, as kairomones to facilitate host localization and mating [129].

Host aggregation pheromones are also exploited by parasitoids for host localization. For example, the male bean bug *Riptortus clavatus* (Heteroptera: Alydidae) releases (*E*)−2-hexenyl (*Z*)−3-hexenoate as a component of its aggregation pheromone, which attracts both conspecifics and the parasitoid *Ooencyrtus nezarae* (Hymenoptera: Encyrtidae), significantly increasing the parasitism rates of *R. clavatus* eggs under field conditions [130, 131]. Behavioral assays via a four-arm olfactometer revealed that the aggregation pheromone *Rhyzopertha dominica* (Coleoptera: Bostrichidae) is highly attractive to the parasitoid *Lariophagus distinguendus* (Hymenoptera: Pteromalidae) [132]. Additionally, *Halticoptera rosae* (Hymenoptera: Pteromalidae) uses the pheromone marking trail of its host, *Rhagoletis basiola* (Diptera: Tephritidae), to locate the fly’s eggs [133].

#### *Hostadultscales*

Many parasitoids target lepidopteran insects as hosts, using compounds at the host scale as cues for host localization. Lewis et al. (1972) demonstrated through greenhouse and field behavioral experiments that hexane extracts of adult *Cadra cautella* (Lepidoptera: Pyralidae) moth scales significantly increased the parasitism efficiency of *Trichogramma evanescens* (Hymenoptera: Trichogrammatidae) on *C. cautella* eggs, providing the first evidence that host scales serve as a signal for parasitoid host localization [134]. Subsequently, Lewis et al. (1973) identified four active compounds from hexane extracts of adult *Helicoverpa zea* (Lepidoptera: Noctuidae) scales—docosane, tricosane, tetracosane, and pentacosane—which increased the parasitism efficiency of *T. evanescens* on *H. zea* eggs, with tricosane exhibiting the most pronounced effect [135]. Additionally, Fatouros et al. (2005) reported that scales deposited on Brussels sprout plants by the butterfly *Pieris brassicae* (Lepidoptera: Pieridae) attracted the egg parasitoid Trichogramma brassicae (Hymenoptera: Trichogrammatidae) [136].

##### Host feces

In the 1970s, chemical ecologists identified volatile compounds from host feces as key signals for parasitoid host localization. Jones et al. (1971) demonstrated through choice experiments in Petri dishes (9 cm diameter) that *Microplitis croceipes* (Hymenoptera: Braconidae) is attracted to 13-methylhentriacontane, a compound in the feces of its host, *H. zea* larvae, marking the first reported insect kairomone associated with parasitic wasp behavior [137]. Similarly, behavioral assays revealed that *Orgilus lepidus* (Hymenoptera: Braconidae) exhibited a significant preference for filter paper treated with n-heptanoic acid, a fecal component of *Phthorimaea operculella* (Lepidoptera: Gelechiidae) larvae [138]. Comparative studies revealed that *Cotesia rubecula* (Hymenoptera: Braconidae) strongly preferred the feces of its primary host, *P. rapae*, over that of its secondary host,* P*. *brassicae*, but showed no preference for the feces of the non-host *Pieris napi* (Lepidoptera: Pieridae) second-instar larvae, indicating that host feces serve as an effective host localization signal [139]. Additionally, (*Z*,*Z*)−6,9-heptacosadiene, a fecal component of *Periplaneta americana*, is highly attractive to *Aprostocetus hagenowii* [140]. Furthermore, allyl isothiocyanate, which is isolated from the feces of second-instar *P. xylostella* larvae, strongly attracted *T. chilonis* and *C. plutellae* [109]. As a marker compound released by cruciferous plants, allyl isothiocyanate suggests that the attractiveness of host feces to parasitoids is partly derived from plant-associated compounds.

##### Uncommon host-derived signals

Parasitoids exploit unique chemical cues directly emitted by their hosts to locate and discriminate suitable targets. For instance, the parasitoid *Diachasmimorpha longicaudata* (Hymenoptera: Braconidae) significantly stimulates oviposition behaviors at close range (< 1 m) in response to para-ethylacetophenone (p-EAP), a major volatile kairomone released by larvae of four Tephritidae species but absent in non-tephritid fly larvae, serving as a reliable olfactory cue for host location despite its low detectability [141].

Mandibular gland secretions of lepidopteran larvae also serve as reliable host-location signals. For example, 2-acylcyclohexane-1,3-diones, which are secreted by the mandibular glands of fifth-instar *Ephestia kuehniella* (Lepidoptera: Pyralidae) larvae, elicit an arrestment, antennation, and probing response when present in a patch and a trail-following response when deposited linearly in *Bracon hebetor* (Hymenoptera: Braconidae) [142]. Moreover, long-chain hydrocarbons (32-, 33-, and 34-carbon saturated hydrocarbons) from the mandibular glands of *Heliothis virescens* (Lepidoptera: Noctuidae) larvae attract *Cardiochiles nigriceps* (Hymenoptera: Braconidae), significantly increasing the antennal probing frequency on treated filter papers [143].

Honeydew, a byproduct of sap-feeding insects, also functions as a kairomone, mediating host location by parasitoids [144]. For example, honeydew from the pistachio psylla *Agonoscena pistaciae* (Hemiptera: Psylloidea) acts as both a volatile and contact kairomone, enhancing the search behavior of the encyrtid parasitoid *Psyllaephagus pistaciae* (Hymenoptera: Encyrtidae) [145]. Furthermore, *Encarsia bimaculata* (Hymenoptera: Aphelinidae) uses honeydew from *Bemisia tabaci* (Hemiptera: Aleyrodidae) as a contact kairomone to locate its host [146]. Similarly, volatiles from the honeydew of the greenhouse whitefly *Trialeurodes vaporariorum* (Homoptera: Aleyrodidae) attract *Encarsia formosa* (Hymenoptera: Aphelinidae) [147]. Recent studies have identified 1-ethyl-2-methylbenzene and 2-butyl-1-octanol, which are emitted by the bacterium *Lysinibacillus fusiformis*, as attractants for the aphid parasitoid *Aphidius gifuensis* (Hymenoptera: Braconidae), suggesting that the honeydew-associated microbiota contributes to the kairomonal activity of honeydew [148].

Cornicle secretions from aphids serve as contact kairomones for several parasitoids. The braconid *Lysiphlebus testaceipes* (Hymenoptera: Aphidiidae) increases the attack frequency in response to cornicle wax secretions from its host *Rhopalosiphum padi* (Homoptera: Aphididae) [149]. Additionally, cornicle secretions from the aphid *Acyrthosiphon pisum* (Homoptera: Aphididae) act as a contact kairomone for the parasitoid *Aphidius ervi* (Hymenoptera: Braconidae), aiding in host recognition and acceptance by females [150]. These secretions, along with cuticular compounds, also contribute to host recognition by *A. ervi* females [151]. Additionally, hemolymph components, such as hexoses and amino acids, from *Galleria mellonella* (Lepidoptera: Pyralidae) and *Celerio euphorbiae* (Lepidoptera: Sphingidae) stimulate parasitic behavior in *Itoplectis conquisitor* (Hymenoptera: Ichneumonidae) [152].

### Semiochemicals related to superparasitism

Hyperparasitoids may exploit the above-mentioned chemical cues to locate already-parasitized hosts, resulting in interspecific superparasitism. This process dampens trophic cascades and reduces the efficacy of biological control against herbivorous pests. For example, the hyperparasitoid *Lysibia nana* (Hymenoptera: Ichneumonidae) uses HIPVs, such as DMNT and 2,4-pentadienenitrile, which are emitted in response to feeding by *P. rapae* caterpillars parasitized by the primary parasitoid *C. glomerata* [153–155]. In addition to using HIPVs for long-range host location, hyperparasitoids may use cues emitted directly by the herbivorous host upon arrival on the infested plant. For example, the hyperparasitoid *Baryscapus galactopus* (Hymenoptera: Eulophidae) is attracted to the body volatiles of *P. rapae* larvae parasitized by *C. glomerata*, with attraction potentially mediated by 3-octanone, (*Z*)−3-hepten-1-ol, 3-methyl-3-buten-1-ol, 3-octanol, and 3-pentanone [156, 157]*.* Hyperparasitoids may also exploit cues associated with their primary parasitoid hosts, such as honeydew [158]. Furthermore, hyperparasitoids can use habitat-related cues to locate primary parasitoids; for example, the hyperparasitoid *Dendrocerus aphidum* (Hymenoptera: Megaspilidae) exploits monoterpenes released by bacteria associated with the habitat of its primary parasitoid host, *A. colemani* [159].

## Host counter-adaptation strategies

Although parasitoids are efficient hunters, they are not invariably successful in every attack. Through co-evolution, hosts have developed a kaleidoscope of countermeasures to evade or resist parasitism. These host strategies are broadly classified into three categories: (1) avoidance and concealment; (2) behavioral and morphological defenses; and (3) physiological defenses [160]. It is well known that host larvae and adults detect HIPVs to avoid feeding and oviposition on plants that emit these “cry for help” signals. For example, *S. littoralis* exhibits significantly reduced oviposition on herbivore-infested cotton plants and even avoids plants adjacent to damaged plants under both field and laboratory conditions [161, 162]. To counter egg parasitoids, many insects, including *Spodoptera* spp., deposit egg masses within protective oothecae or coverings composed of scales, setae, silk, or spumaline. These structures create physical barriers that impede ovipositor penetration by parasitoids and suppress the induction of OIPVs [160, 163]. To thwart larval parasitoids, host larvae deploy a suite of evasive behaviors upon detecting an imminent attack, including vigorous wriggling, thrashing, rolling, curling, biting, and even leaping [160]. Moreover, larvae sequester plant secondary metabolites via their oral regurgitant and spit them as an anti-parasitoid defence [164]. Notably, certain parasitoids have evolved to exploit this regurgitant as a kairomonal cue; for example, *M. croceipes* is strongly attracted to the regurgitant of *H. virescens* [165]. To resist pupal parasitoids, lepidopteran pupae execute vigorous wriggling and rotational movements that deflect the ovipositors of the attacking parasitoids [166]. Once parasitized, host insects mobilize cellular immune responses that can encapsulate and kill parasitoid eggs [167]. Recent studies have revealed that dinidorid stinkbugs possess a conspicuous tympanal organ on female hindlegs. This organ is no longer used for auditory perception but has been co-opted to harbor fungal symbionts, thereby protecting eggs from parasitization by wasps [168]. These studies highlight the “tug-of-war” between parasitoids and their hosts.

## Detection of semiochemicals by parasitoids

Insects rely on olfaction and gustation to detect external chemical stimulants, which are mediated by specialized chemosensory receptors. Olfactory receptors, including odorant receptors (ORs), ionotropic receptors (IRs), and CO_2_ receptors, are critical for odor recognition, whereas gustatory receptors (GRs) primarily facilitate gustation [169–171]. In parasitoids, the OR gene family has notably expanded, reflecting the critical role of olfaction in their ecology. For example, *Nasonia vitripennis* (Hymenoptera: Pteromalidae) has 301 ORs [172], *C. chlorideae* has 211 ORs [84], *M*. *mediator* has 177 ORs [173]*, Trichogramma pretiosum* (Hymenoptera: Trichogrammatidae) has 105 ORs [174], *Trissolcus basalis* (Hymenoptera: Scelionidae) has 170 ORs [175], and *Anastatus japonicus* (Hymenoptera: Eupelmidae) has 184 ORs [176]. This high OR diversity underscores the importance of olfaction in parasitoid host-seeking behavior.

However, despite the abundance of chemosensory receptors in parasitoids, most remain uncharacterized ("orphan" receptors), hindering a comprehensive understanding of how parasitoids locate mates and suitable hosts precisely. To address the “detectability‒reliability” dilemma in host locations, parasitoids likely employ distinct chemosensory gene classes at different stages of host searching. During habitat searching, environmental olfactory cues are highly detectable but less reliable. Parasitoids may utilize less abundant ORs and IRs with broad-tuning profiles to detect these general habitat-related signals. As parasitoids approach their hosts, they likely rely on narrowly tuned receptors to detect reliable cues, such as HIPVs and host-derived chemicals.

**Fig. 1:**
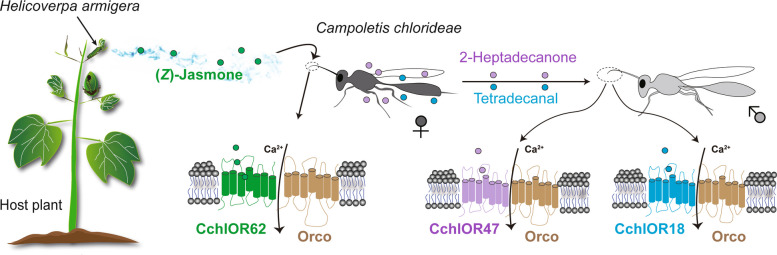
Olfactory detection mechanisms for (*Z*)-jasmone and sex pheromones in *Campoletis chlorideae* (Hymenoptera: Ichneumonidae). OR: odorant receptor; Orco: odorant receptor coreceptor

Recent studies have begun to elucidate the molecular basis of olfaction in parasitoids, particularly through the identification of ORs and IRs tuned to specific chemical cues. In *C. chlorideae*, two male antennae-expressed ORs, CchlOR18 and CchlOR47, were identified as receptors for the sex pheromones tetradecanal and 2-heptadecanone, respectively, marking the first characterized sex pheromone receptors in parasitoids [48] (Fig. 1). Additionally, CchlOR62, which is exclusively expressed in female antennae, is tuned to (*Z*)-jasmone, a HIPV from cotton and tobacco plants that strongly attracts female *C. chlorideae* [84] (Fig. 1)*.* Interestingly, CchlOR18, CchlOR47, and CchlOR62 are narrowly tuned receptors, suggesting that parasitoids employ highly selective ORs to detect reliable but less detectable compounds, such as sex pheromones and HIPVs.

In *M. mediator*, MmedOR49 is specifically tuned to the host sex pheromone *cis*−5-decenyl acetate (*Z*5-10:Ac), providing molecular evidence that parasitoids exploit host sex pheromones as kairomones for host location [177]. Moreover, MmedOR48, a broadly tuned OR, responds to 23 compounds, including five plant aldehyde volatiles, indicating its role in detecting general habitat cues [178]. In *A. japonicus*, a parasitoid of the litchi pest *Tessaratoma papillosa* (Heteroptera, Pentatomidae), AjapOR35 was identified as a detector of the oviposition attractants *β*-caryophyllene and (*E*)-*α*-farnesene through RNAi functional screens [89]. Conversely, ORs tuned to repellent cues have also been reported. In *Baryscapus dioryctriae* (Hymenoptera: Eulophidae), BdioOR58 is selectively tuned to 1-octen-3-ol, a compound that repels this parasitoid [179].

With respect to IRs, in *M. mediator*, MmedIR64a1 is broadly tuned to habitat-related chemical cues from host plants, facilitating long-distance host searching, whereas MmedIR64a2 responds narrowly to low-volatility host cues and plant odors, including *Z*9-14:Ald, a minor sex pheromone component of *H. armigera* [123]. These findings reinforce the hypothesis that parasitoids use narrowly tuned receptors for highly reliable, low-detectability cues, such as host sex pheromones, whereas broadly tuned receptors detect general environmental signals.

Systematic functional characterization of ORs and IRs is essential to fully elucidate how parasitoids exploit olfactory cues of varying reliability and detectability for host location. However, the extensive diversity of olfactory receptors in parasitoids hinders a comprehensive understanding of their olfactory mechanisms. Despite the large receptor repertoire and the vast number of parasitoid species, ORs and IRs remain “orphan” in most parasitoids. Functional characterization has advanced in two larval endoparasitoids of cotton bollworms, *C. chlorideae* and *M. mediator*, yet only a handful of receptors have been deorphanized to date [48, 84, 123, 178]. In commercially important genera such as *Trichogramma* spp. and *Telenomus* spp., no OR functions have been reported, likely due to the minute body size of these parasitoids, which poses significant challenges for behavioral monitoring and physiological manipulation.

In parasitoids, gustation plays a critical role in detecting contact cues associated with mates, hosts, and food sources [180, 181]. However, the functions of GRs in parasitoids remain underexplored. In the egg parasitoid *T. chilonis*, two GRs, TchiGR64f1 and TchiGR64f2, when co-expressed in *Xenopus* oocytes, respond to sucrose, whereas TchiGR43a, when expressed alone, responds to D-fructose [182, 183]. These sugar-responsive GRs are likely involved in locating food sources, such as floral nectar [184–186]. In contrast, the GRs responsible for mate and host localization remain poorly understood. Notably, the detection of non-volatile cuticular chemicals by GRs is likely a key step in mate recognition [48]. Additionally, the presence of gustatory organs on the ovipositor of parasitoids suggests that certain GRs are expressed in ovipositor sensilla, potentially mediating the detection of chemical cues critical for host discrimination [187, 188]. Thus, elucidating the functions of GRs is essential for obtaining a comprehensive understanding of parasitoid biology.

## Manipulation of parasitoid behaviors via synthetic semiochemicals

Decades of laboratory research on semiochemicals have opened new avenues for manipulating parasitoid behaviors to enhance pest management. Laboratory-based experiments, such as olfactometer tests, oviposition assays, and cage studies, have been instrumental in evaluating the effects of semiochemicals on parasitoid behavior. However, these methods often fail to capture the dynamic nature of odor plumes under field conditions, highlighting the need for reliable field-based platforms to assess their semiochemical efficacy.

Synthetic biology, which involves designing and engineering biological systems for novel functions [189], offers promising solutions for the consistent production and release of semiochemicals in the field, overcoming limitations of natural induction, such as inconsistent emission or reliance on herbivore damage [190]. By engineering plant or yeast metabolic pathways, synthetic biology enables these organisms to serve as chassis for producing specific semiochemicals, including HIPVs, host alarm pheromones, and host sex pheromones, which function as synomones and kairomones to attract parasitoids, thereby increasing their recruitment and efficacy against herbivorous pests (Table 3) [212].

**Table 3:** Biosynthesis of plant volatiles in transgenic plants

Transgenic plants	Expressed genes	Products	Behavioral effects	References
*Arabidopsis thaliana*	*EβfS* from peppermint (*Mentha* × *piperita*)	(*E*)-*β*-farnesene	Repelling aphid *Myzus persicae* and retaining parasitoid *Diaeretiella rapae*	[124]
*Arabidopsis thaliana*	*FPS2 from Arabidopsis thaliana*	Sesquiterpenes, including (*E*)-*β*-farnesene	Triggering agitation in aphid *Myzus persicae*	[191]
*Arabidopsis thaliana*	*FaNES1* from strawberry	(*E*)-nerolidol DMNT Linalool	Repelling aphid *Brevicoryne brassicae* and attracting parasitoid *Diaeretiella rapae*	[192]
*Arabidopsis thaliana*	*TPS10* from maize *Zea mays*	(*E*)-*β*-farnesene (*E*)-*α*-bergamotene	Attracting parasitoid *Cotesia marginiventris*	[193]
*Arabidopsis thaliana*	*TPS8* from maize *Zea mays*	α –copaene; (*E*)-*β*-caryophyllene; germacrene D; *δ*-cadinene	Attracting parasitoid *Cotesia marginiventris*	[194]
*Nicotiana tabacum*	*GhCYP82Ls* and *GhTPS14* from cotton *Gossipium hirsutum*	DMNT	Attracting parasitoid *Microplitis mediator*	[195]
*Nicotiana tabacum*	*AaβFS1* from sweet wormwood *Artemisia annua*	(*E*)-*β*-farnesene	Repelling aphid *Myzus persicae* nd attracting parasitoid *Diaeretiella rapae*	[196]
*Nicotiana tabacum*	*MaβFS1* and *MaβFS2* from peppermint (*Mentha* × *piperita*)	(*E*)-*β*-farnesene	Repelling aphid *Myzus persicae* and attracting predator *Chrysopa septempunctata*	[197]
*Nicotiana tabacum*	*β-ocimene synthase* from lima bean	*β*-ocimene	Repelling aphid *Macrosiphum euphorbiae* and attracting parasitoid *Aphidius ervi*	[198]
*Nicotiana tabacum*	*HMGR* and* santalene synthase*	Santalene; Bergamotene	Attracting aphid *Myzus persicae*	[199]
*Nicotiana tabacum*	*Isoprene synthase* from white poplar *Populus alba*	Isoprene	Repelling pest *Manduca sexta*	[200]
*Nicotiana tabacum*	*Monoterpene synthases* from lemon *Citrus limon*	*β*-pinene Limonene *γ*-terpinene	Not investigated	[201]
*Gossypium hirsutum*	*GhCYP82Ls* and *GhTPS14* from cotton *Gossypium hirsutum*	DMNT	Attracting mirid bug *Apolygus lucorum* and parasitoid *Peristenus spretus*	[202]
*Gossypium hirsutum*	*GhTPS16* from cotton *Gossypium hirsutum*	(*E*)-*β*-ocimene	Attracting parasitoids *Apolygus lucorum* and *Microplitis mediator*	[203]
*Gossypium hirsutum*	*GhTPS1* from cotton *Gossypium hirsutum*	(*E*)-*β*-caryophyllene	Repelling pests and attracting parasitoids *Peristenus spretus* and *Aphidius gifuensis*	[204]
*Zea mays*	EβC synthase *tps6* from oregano *Origanum vulgare*	(*E*)-*β*-caryophyllene	Attracting insect-killing nematode *Heterorhabditis megidis*	[205]
*Oryza sativa*	*OsTPS3* from rice *Oryza sativa*	(*E*)-*β*-caryophyllene	Attracting *Anagrus nilaparvatae*	[206]
*Oryza sativa*	*EβFS from black peppermint*	(E)-*β*-farnesene	Repelling aphid *Rhopalosiphum padi*	[207]
*Medicago sativa*	AaEβF from sweet wormwood *Artemisia annua*	(*E*)-*β*-farnesene	Repelling aphid *Myzus persicae*	[208]
*Triticum aestivum*	*EβfS* and *FPPS* from peppermint (*Mentha* × *piperita*)	(*E*)-*β*-farnesene	Repelling aphids and attracting parasitoid *Aphidius ervi*	[209]
*Brassica juncea*	*EβfS* from peppermint (*Mentha* × *piperita*)	(*E*)-*β*-farnesene	Repelling aphid *Lipaphis erysimi*	[210]
*Lotus japonicus*	*PlTPS2* from lima bean *Phaseolus lunatus*	TMTT	Attracting predator *Neoseiulus californicus*	[211]

DMNT represents (3*E*)−4,8-dimethyl-1,3,7-nonatriene; TMTT represents (*E*,*E*)−4,8,12-trimethyl-1,3,7,11-tridecatetraene

Recent advances have demonstrated the synthesis of terpenoids, primarily mono- and sesquiterpenes, in model plant "factories" through genetic engineering with terpene synthase (TPS) genes, resulting in stable emission and attraction of parasitoids [213]. This approach strengthens tritrophic interactions (plant–herbivore–parasitoid interactions) and supports sustainable integrated pest management (IPM) [214]. A notable example is (*E*)-*β*-farnesene (EβF), a multifaceted sesquiterpene that acts as a HIPV in certain plants, an alarm pheromone for aphids, and a kairomone for natural enemies [215, 216]. EβF synthase genes, which encode enzymes that convert farnesyl diphosphate (FPP) to EβF, have been isolated and characterized in species such as Douglas fir, yuzu, sweet wormwood, and black peppermint, making EβF a prime candidate for transgenic expression in model plants to test synthetic biology approaches for pest control [217]. Accordingly, EβF has been transgenically produced in *Arabidopsis thaliana* [124, 191], tobacco (*Nicotiana tabacum*) [196, 197], alfalfa (*Medicago sativa*) [208], rice (*Oryza sativa*) [207], wheat (*Triticum aestivum*) [209], and mustard (*Brassica juncea*) [210].

*Arabidopsis thaliana* and *N. tabacum* have served as robust model systems for pioneering terpenoid metabolic engineering [218, 219]. For example, *A. thaliana* engineered to express the peppermint (*Mentha* × *piperita*) (*E*)-*β*-farnesene synthase gene (EβfS) produces EβF, eliciting strong alarm and repellent responses in the aphid *M. persicae* while attracting and retaining the parasitoid *D. rapae* (Hymenoptera: Braconidae) [124]. Similarly, *A. thaliana* overexpressing the maize terpene synthase gene *TPS10* emits a sesquiterpene blend, including (*E*)-*β*-farnesene and (*E*)-*α*-bergamotene—HIPVs released by maize under herbivore attack—thereby attracting the parasitoid *C. marginiventris* [193]. Additionally, transgenic *A. thaliana* expressing the strawberry linalool/nerolidol synthase gene (*FaNES1*) emits elevated levels of (*E*)-nerolidol, (*E*)-DMNT, and linalool, repelling the aphid *Brevicoryne brassicae* (Homoptera, Aphididae) and attracting the parasitoid *D. rapae* [192].

In the tobacco *N. tabacum*, engineering with three monoterpene synthase genes from *Citrus limon* significantly increased the emission of monoterpenoids, including *β*-pinene, limonene, and *γ*-terpinene, by 10- to 25-fold in leaves and flowers compared with that of wild-type plants [201]. Expression of the sweet wormwood (*Artemisia annua*) EβF synthase gene (*AaβFS1*) in tobacco chloroplasts, mediated by the chloroplast transit peptide (CTP) of the wheat *T. aestivum,* results in EβF emission and attraction of the parasitoid *D. rapae* [196]. These findings suggest that chloroplasts are an ideal subcellular compartment for engineering plant-derived EβF synthase genes to develop novel transgenic plants for aphid control. Furthermore, overexpressing a cotton terpene synthase gene for (*E*)-*β*-ocimene biosynthesis in *N. tabacum* recruits parasitoids such as *Peristenus spretus* (Hymenoptera: Braconidae) and *M*. *mediator*, indicating that (*E*)-*β*-ocimene is a promising candidate for novel IPM strategies [203].

Cotton plants have also been utilized in synthetic biology to express new plant volatiles. For example, cotton expressing two DMNT-synthesis genes (*GhCYP82L1* and *GhTPS14*) emits 10- to 15-fold higher DMNT levels than wild-type controls do, significantly increasing the recruitment of *P*. *spretus* [220]. Similarly, *β*-caryophyllene, synthesized and released by cotton plants overexpressing the caryophyllene synthase gene *GhTPS1*, repels pests such as *Aphis gossypii* (Homoptera: aphididae), *A. lucorum*, and *H. armigera* while attracting parasitoids *P. spretus* and *Aphidius gifuensis* (Hymenoptera: Aphidiidae) [204].

In addition to parasitoids, terpenoid release can modulate the behavior of entomopathogenic nematodes, another group of natural enemies. For example, a maize line susceptible to western corn rootworm, which typically lacks (*E*)-*β*-caryophyllene, was engineered to express an oregano (*E*)-*β-*caryophyllene synthase gene, restoring emission and enhancing below-ground defense by attracting entomopathogenic nematodes that infect and kill the root pest [205].

In addition to terpenoids, insect sex pheromones and their precursors have been produced in transgenic plants through the expression of key enzymes involved in pheromone biosynthesis. For example, (*Z*)−11-hexadecenal (*Z*11–16:Ald), (*Z*)−11-hexadecenol (*Z*11–16:OH), and (*Z*)−11-hexadecenyl acetate (*Z*11–16:OAc), which serve as sex pheromone components for various lepidopteran moths [221, 222], have been synthesized in transgenic plants such as tobacco and Camelina and attract pests, including *Mamestra brassicae* (Lepidoptera: Noctuidae) and *P. xylostella* [223–225]. However, no research has been reported on genetically modified plants that produce pheromones from parasitoids.

In addition to transgenic plants, yeast cell factories have been utilized to produce plant volatiles and insect sex pheromones. For example, reconstructing terpenoid synthesis pathways in yeast has enabled the production of citronellol, geraniol, and nerol in ratios closely resembling those emitted by *Rosa* × *damascena* [226]. Furthermore, metabolic engineering of oleaginous yeasts, such as *Yarrowia lipolytica* and *Saccharomyces cerevisiae*, through the expression of fatty acyl-CoA desaturases (*FADs*) and fatty acyl reductases (*FARs*), has facilitated the production of moth sex pheromones [227]. Notable examples include (*Z*)−11-hexadecenal (*Z*11–16:Ald), the primary sex pheromone component of the cotton bollworm *H. armigera* [228]; (*Z*)−9-tetradecenyl acetate (*Z*9-14:OAc), the primary sex pheromone component of the fall armyworm *Spodoptera frugiperda* (Lepidoptera: Noctuidae) [228];* Z*11–14:OAc, the main sex pheromone component of the Z-race of *O. nubilalis* [229]; and *E*10,*Z*12–16:OH, the main sex pheromone component of *Bombyx mori* (Lepidoptera: Bombycidae) [230].

However, the influence of yeast-produced plant volatiles and insect sex pheromones on parasitoid behavior remains largely unexplored. Yeasts, as an in vivo synthesis system, can produce semiochemicals in ratios optimized for target insects via cost-effective substrates [228, 231]. These findings suggest that the synthesis of semiochemicals from yeast factories has significant potential for manipulating parasitoid behavior in biocontrol applications. Future strategies could involve inoculating susceptible plants with transgenic yeasts engineered to emit parasitoid-attractive semiochemicals or directly applying yeast-derived semiochemical extracts to plants to increase parasitoid attraction.

Taken together, the metabolic engineering of plant volatiles has shown significant potential for enhancing indirect plant defense by attracting parasitoids. Notably, transgenic plants producing and emitting (*E*)-*β*-farnesene, (*E*)-*β*-caryophyllene, and (*E*)-*β*-ocimene have demonstrated dual functionality, repelling pests while attracting parasitoids, making them promising candidates for novel "push–pull" strategies (Table 3).

## Enhancement of parasitoid efficacy through companion planting

Companion planting, or intercropping, is a widely adopted habitat manipulation strategy that increases the abundance of natural enemies by providing alternative food sources and shelter [232]. Effective companion plants should increase the fitness of natural enemies, such as parasitoids, without benefiting their hosts or negatively impacting crop growth through competition. This approach has proven effective in "push–pull" strategies [233]. For example, intercropping maize with the molasses grass *Melinis minutiflora* significantly reduces stem-borer infestation and increases larval parasitism by *C. sesamiae* [234]. This effect is attributed to volatiles emitted by *M. minutiflora,* such as DMNT, which repel female stem borers and attract foraging female *C. sesamiae* [234]. Similarly, the cornflower *Centaurea cyanus* is highly attractive to the parasitoid *M. mediator*, enhancing its survival and parasitism rates [235, 236]. Additionally, headspace volatiles from companion plants such as *Desmodium intortum*, *Desmodium uncinatum*, and *Brachiaria Mulato* deter oviposition by the pest *S. rugiperda* on maize while attracting the larval endoparasitoids *Cotesia icipe* (Hymenoptera: Braconidae) and *Coccygidium luteum* (Hymenoptera: Braconidae) [237]. Other companion plants, including greenleaf desmodium, silverleaf desmodium, sweet potato, beans, cassava, and groundnut, also attract *C. icipe* while deterring *S. frugiperda* oviposition on maize [238]. These effects are likely driven by behavior-modifying compounds emitted by companion plants, such as (*Z*)−3-hexenyl acetate, (*E*)-*β*-ocimene, DMNT, (*E*)-*β*-caryophyllene, camphor, methyl salicylate, and TMTT [238].

However, a key challenge of companion planting is the potential to attract additional pests, complicating intercropping strategies. For example, intercropping buckwheat with soybeans significantly reduces populations of pests such as *Riptortus pedestris* (Hemiptera: Alydidae) but increases the density of other pests, such as leafminers and leafhoppers [239].

## Challenges in applying semiochemicals for parasitoid-based pest management

Despite the above advances, field applications of semiochemicals to increase parasitoid efficacy face significant challenges stemming from the complexity of natural environments. For example, deploying the attractant borneol in apple orchards elevated the densities of the egg parasitoid *Cyzenis albicans* (Diptera: Tachinidae), which targets the winter moth *Operophtera brumata* (Lepidoptera: Geometridae), yet the parasitism rates remained unaffected, likely due to the absence of oviposition-determining cues [240]. Similarly, wheat genetically engineered to constitutively emit the aphid alarm pheromone (*E*)-*β*-farnesene repelled aphids and attracted the parasitoid *A. ervi* in olfactometer assays; however, field trials revealed no decline in aphid populations or increase in parasitism [209], potentially resulting from olfactory habituation caused by prolonged odorant exposure [241]. Furthermore, localized aggregation of parasitoids induced by semiochemicals can disrupt population dynamics, rendering adjacent areas more susceptible to pest outbreaks [241]. The novel compounds synthesized in plants may also impair plant growth and development by competing for the limited carbon resources needed for essential metabolites or through direct toxicity [242–244].

Parasitoids are highly sensitive to abiotic stressors, including temperature, humidity, and light. For example, the parasitism of *Trichogramma brassicae* decreases by nearly 40% at temperatures exceeding 35°C [245]. Plants subjected to heat, drought, or elevated ozone (O₃) levels release volatile blends that closely resemble HIPVs, acting as deceptive cues that markedly diminish parasitoid foraging efficiency [246]. Furthermore, atmospheric pollutants—particularly ozone, nitrogen oxides, and hydroxyl radicals—degrade HIPVs, further hindering host location by parasitoids [247]. Thus, both biotic and abiotic factors must be carefully considered when evaluating the field efficacy of semiochemical-based biocontrol strategies.

## Concluding remarks and future perspectives

**Fig. 2:**
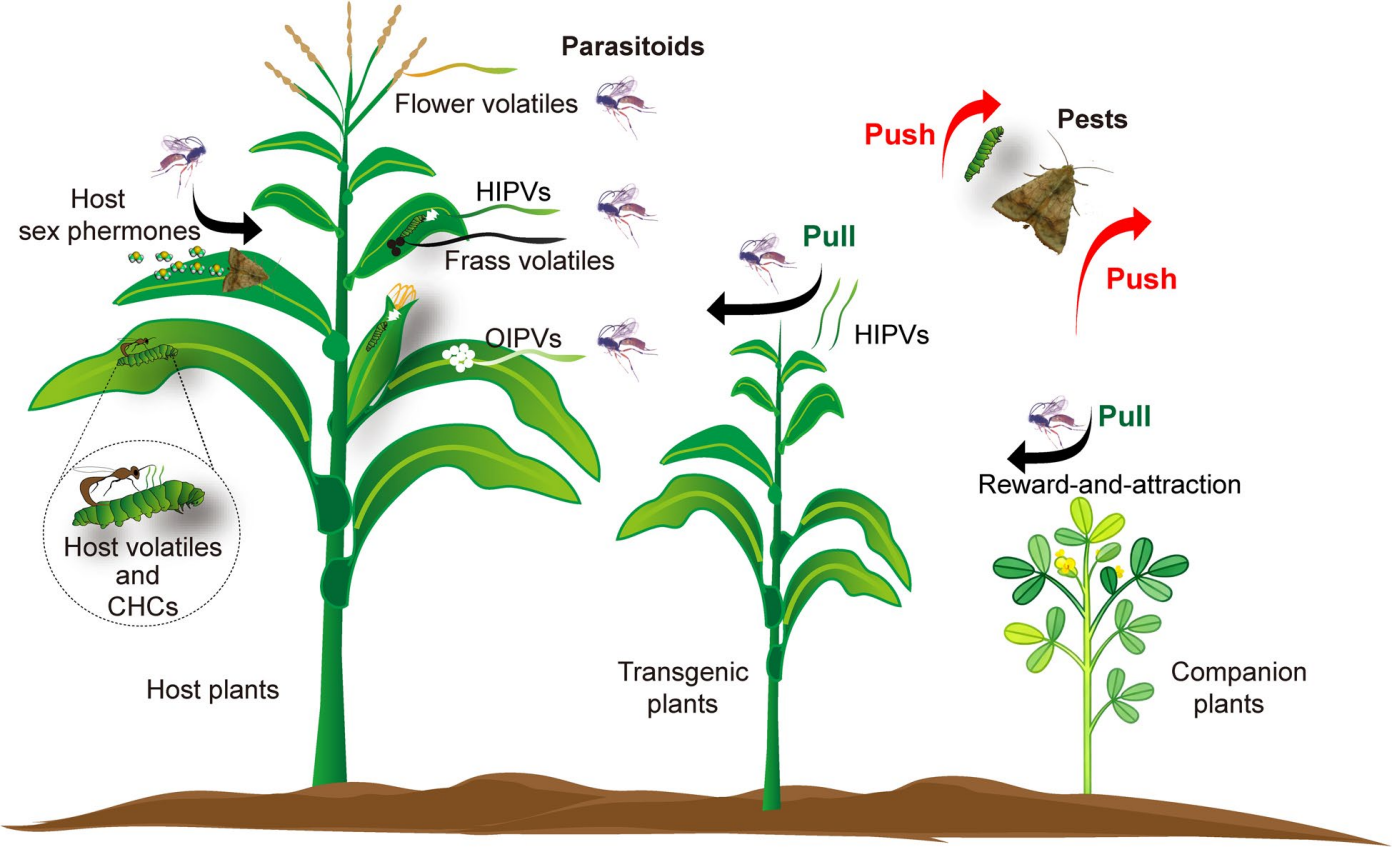
Summary of semiochemicals used by parasitoids for host location and the “push–pull” strategy involving parasitoid attraction and pest repellence. HIPVs (herbivore-induced plant volatiles); OIPVs (oviposition-induced plant volatiles); CHCs (cuticle hydrocarbons)

Over the past few decades, a diverse array of semiochemicals employed by parasitoids for host location has been identified. At a distance, parasitoids integrate multiple cues, such as HIPVs, OIPVs, host frass volatiles, and host sex pheromones, to locate potential hosts. Upon contact, parasitoids assess host suitability by detecting host body volatiles and CHCs (Fig. 2). In field applications, transgenic plants engineered through synthetic biology to express key HIPV components attract parasitoids while repelling pests. Similarly, companion plants protect crops by recruiting parasitoids through reward-and-attraction mechanisms and deterring pests via repellent emissions (Fig. 2). These strategies offer promising avenues for developing innovative “push‒pull” pest management systems.

For biological control, semiochemicals serve multiple purposes: (1) monitoring parasitoid population dynamics to inform artificial release strategies [248]; (2) optimizing mating conditions through sex pheromones to increase mating success and achieve optimal sex ratios in rearing populations for large-scale indoor breeding [249]; and (3) improving host orientation and localization by parasitic wasps, thereby increasing parasitism rates [250]. Despite these advances, several research gaps warrant further investigation to enhance the practical application of semiochemicals in the biological control of pests.

### Synergistic and antagonistic effects of semiochemicals

Plants typically emit complex blends of volatile compounds; however, most studies have focused on single compounds, overlooking potential interactions. For example, (*Z*)-jasmone and (*Z*)−3-hexenyl acetate individually increased the parasitism efficiency of *C. chloridea* on *H. armigera* larvae in indoor cage experiments, but their combination had no effect [85]. Conversely, combining (*Z*)-jasmone with sex pheromones attracts female and male *C. chloridea*, significantly improving host location and mating success, thereby increasing parasitic efficiency [48, 84]. Future research should prioritize the synergistic and antagonistic interactions among volatile blends to optimize their use in pest control.

### Complex trophic interactions

The interactions among plants, pests, and parasitoids are highly intricate. For example, indole, a HIPV released by maize, attracts the parasitoid *M. rufiventris* but also modifies the volatile profile on the body surface of *Spodoptera littoralis* (Lepidoptera: Noctuidae) larvae, reducing their attractiveness to the parasitoid and lowering parasitism rates [251]. Furthermore, *S. littoralis* larvae, which typically avoid indoles, cease to do so in the presence of parasitoids, suggesting that pests may exploit HIPVs for self-protection [251]. Additionally, hyperparasitoids can exploit plant-emitted synomones and host-derived kairomones to locate and parasitize primary parasitoids, undermining their efficacy in pest control [252]. These complex dynamics, including the often overlooked role of hyperparasitism, require greater attention in experimental designs.

### Disorientation effects

The semiochemical redundancy hypothesis posits that high local chemical diversity and concentrations may disrupt herbivore foraging through chemical disorientation and masking [253, 254]. This disorientation may similarly impair the ability of specialized natural enemies, such as parasitoids, to locate their prey [255, 256]. Future research should focus on designing indoor and field experiments to disentangle the effects of odorant disorientation on parasitoid behavior, providing insights into optimizing semiochemical-based strategies.

### Translation of laboratory findings to field applications

Current research on the regulatory effects of semiochemicals on parasitoid behavior is largely confined to laboratory or semi-field studies. To date, no commercially available parasitoid attractants have been developed. Future efforts should prioritize large-scale field experiments to validate laboratory findings under real-world conditions. To bridge this gap, experiments should incorporate real plants and large-scale greenhouse setups to simulate field conditions, ensuring that the results are more applicable to practical pest management.

### Advancing semiochemical delivery methods

The development of effective methods for releasing semiochemicals in the field is critical. Traditional approaches, such as spraying or slow-release lures, are resource intensive and, if improperly applied, may cause disorientation. The use of transgenic plants engineered to emit key semiochemicals represents a promising solution, particularly with advancements in gene-editing technologies.

### Identifying active compounds via reverse chemical ecology

Parasitoid genomes harbor numerous OR genes, most of which remain uncharacterized. In particular, the olfactory detection mechanisms of commercially used species, such as *Trichogramma* spp. and *Telenomus* spp., remain completely unknown, hindering the identification of effective semiochemicals to increase their parasitism efficacy. Recent advances in machine learning, such as quantitative structure‒activity relationship (QSAR) modeling, have achieved up to 95% success in predicting ligands for lepidopteran ORs [257]. The application of QSAR to predict ligands for parasitoid ORs, followed by functional validation, provides a rapid approach for identifying novel semiochemicals for pest control.

## Data Availability

Not applicable.
